# Workplace Lactation Support: A Cross-Sectional Study in a University Hospital and a Perinatal Network

**DOI:** 10.3390/nu14173463

**Published:** 2022-08-24

**Authors:** Chloé Barasinski, Marina Stankovic, Anne Debost-Legrand, Amélie Delabaere, Françoise Vendittelli, Frédéric Dutheil

**Affiliations:** 1 Centre Hospitalier Universitaire (CHU) Clermont-Ferrand, Centre National de la Recherche Scientifique (CNRS), Institut Pascal, Université Clermont Auvergne, F-63000 Clermont-Ferrand, France; 2 School of Midwifery, University of Clermont-Auvergne, F-63000 Clermont-Ferrand, France; 3 CHU Clermont-Ferrand, CNRS, Institut Pascal, Réseau de Santé en Périnatalité d’Auvergne, Université Clermont Auvergne, F-63000 Clermont-Ferrand, France; 4 CNRS, Laboratoire de Psychologie Sociale et Cognitive (LaPSCo), Physiological and Psychosocial Stress, CHU Clermont-Ferrand, Occupational and Environmental Medicine, Université Clermont Auvergne, WittyFit, F-63000 Clermont-Ferrand, France

**Keywords:** breastfeeding, health worker, lactation room, policy, return to work, women’s health, working mothers, workplace

## Abstract

Return to work negatively affects the initiation and duration of breastfeeding. Our study’s objective was to assess the percentage of departments in Auvergne with an appropriate space for pumping milk at work. Our cross-sectional survey investigated the arrangements for facilitating the continuation of breastfeeding on return to work at all departments at the Clermont-Ferrand University Hospital Center and perinatal (obstetric and pediatric) departments in this region. Our principal endpoint was the percentage of departments reporting that they had a lactation room—a room where nursing mothers can express milk—and whether it met the criteria defined by the French Labor Code. Among 98 respondents, 44 departments (44.9%) did not offer lactation rooms; of the remaining 54 departments, only 11 rooms met the legal requirements. All perinatal departments offered lactation rooms. The availability of a lactation room was associated with other breastfeeding support, such as a break period for expressing milk (*p* < 0.0001) and the availability of a refrigerator to store it (*p* = 0.01). Almost half the responding departments did not offer a lactation room where mothers could breastfeed or pump their milk. Measures must be envisioned to facilitate the pumping of breast milk by French women returning to work.

## 1. Introduction

Breastfeeding is the method recommended worldwide for nourishing newborns. Beyond its nutritional virtues and its perfect correspondence to babies’ needs, breastfeeding not only has an important positive impact on the infant’s health that continues into childhood but also favorably affects the woman’s health [1]. A large meta-analysis indicates that it protects against infections and malocclusion in children and is associated with higher intelligence and probable reductions in overweight and diabetes. Breastfeeding also safeguards against breast cancer and improves birth spacing. It may also protect against ovarian cancer and type 2 diabetes [2]. Hence, the World Health Organization (WHO) recommends “exclusive breastfeeding for the first 6 months of life and introduction of nutritionally-adequate and safe complementary (solid) foods at 6 months together with continued breastfeeding up to 2 years of age or beyond” [3]. WHO has specified six global nutrition targets for 2025, one of which is to increase the rate of exclusive breastfeeding in the first 6 months up to at least 50% [4]. In France, a recent study reports that recommended feeding practices were related to higher maternal age and education level, so was migration status, the presence of older children, low income, or the mothers’ attendance of pre-birth preparation classes [5].

The initiation and continuation of breastfeeding in industrialized nations varies massively between countries. One of its major obstacles is that women return to work [6,7]. A large French cohort study showed that the older the infant at the mother’s return to work, the greater the likelihood that she had initiated breastfeeding and the longer its duration [8]. A recent systematic review shows that interventions at the workplace are important in protecting, promoting, and supporting breastfeeding among working mothers [9]. In the USA, another review showed that workplace lactation interventions increased breastfeeding initiation, duration, and exclusive breastfeeding [10]. Thus, removing or reducing this obstacle appears likely to improve these rates. The breastfeeding rate in France is one of the lowest in Europe; recent reports show that while the initiation rate is about 72%, breastfeeding decreases rapidly, with less than 25% of infants still breastfed at 6 months [11].

There are, to our knowledge, no scientific publications on existing measures in France to promote the continuation of breastfeeding on return to work. According to a 2014 survey by the French Coordination for Breastfeeding (CoFAM) of mothers who pumped milk at the workplace, 43% found it difficult or very difficult to do so, and only one-third had a comfortable place available for this task; the other mothers had to express their milk in toilets, open spaces, etc. [12]. The French Labor Code nonetheless calls for some measures to support breastfeeding. It requires that nursing mothers have available an hour a day at work to express their milk or breastfeed their infants. Moreover, all companies with more than 100 employees must make a dedicated lactation room available to their employees who wish to breastfeed [13].

The principal objective of our study was to assess the number of departments with an adequate lactation room for pumping milk at work in a University Hospital and in perinatal (that is, obstetric and pediatric) departments across Auvergne belonging to its regional perinatal network. The secondary objectives were to describe the arrangements offered to women to express their milk on their return to work and to assess whether these arrangements differ according to either availability of a lactation room or the type of department.

## 2. Materials and Methods

### 2.1. Design

We conducted a cross-sectional organizational audit about the policies and dispositions reported available to facilitate the continuation of breastfeeding on return to work in the departments at the University Hospital of Clermont-Ferrand and in all obstetrics and pediatrics units belonging to the Auvergne perinatal health network (RSPA).

### 2.2. Materials

In our convenience sample, all of the people responsible for supervising a department at the Clermont-Ferrand University Hospital (regardless of their official status, *n* = 150) as well as all those responsible for supervising an obstetric or pediatric department involved in the RSPA (*n* = 23) were eligible. In 2020, the Clermont-Ferrand University Hospital, the only university hospital in the Auvergne region, had 8153 employees, and the perinatal departments of the RSPA all together employed approximately 1100 staff members (source: Annual Statistics for Health Care Facilities, www.sae-diffusion.sante.gouv.fr, accessed on 6 April 2022).

### 2.3. Development and Pretesting of the Questionnaire

The questionnaire was constructed from various sections of the French Labor Code [13,14] and from the specifications of a U.S. program aimed at companies supporting breastfeeding for women returning to work, the “Texas Mother-Friendly Worksite Program (MFWP)”, which certifies companies as “breastfeeding-friendly” in Texas [15]. To validate its relevance and adapt its contents to the French context, we interviewed 2 experts aiding nursing mothers after their return to work, that is, a lactation consultant and a volunteer midwife at Lact’écoute (an association of volunteers running a breastfeeding hotline in the region around Clermont-Ferrand), as well as 2 occupational physicians working at the Clermont-Ferrand University Hospital and a nurse/quality engineer and a public health quality engineer, also employed there.

The online questionnaire was tested by 4 healthcare managers to validate its understandability and its feasibility.

The questionnaire included a maximum of 71 questions, most of them closed-ended (Questionnaire S1). The questionnaire was divided into 7 parts: (1) outfitting lactation rooms, (2) break time, (3) institutional policy adapted to the department, (4) support measures, (5) education around breastfeeding, (6) description of the respondent’s department, and (7) questions about the respondent. It included a part for free comments and was accompanied by a leaflet explaining the study objectives and detailing the approvals obtained for its performance.

### 2.4. Recruitment Process

The study began on 28 January 2021 and ran through 30 July 2021. Supervisors of departments at the Clermont-Ferrand University Hospital and of RSPA perinatal departments were contacted by email sent by the management of the University Hospital care departments or of its human resources office or by a midwife coordinator of the RSPA for the departments concerned. A reminder email was sent 2 months after the first email. The questionnaire was also distributed via the hospital newsletter.

The study participants were informed on the survey home page of the time required to complete the survey, their right to withdraw from the study (via a personal number obtained at the end of the study), and the study’s objectives. No identifying information was collected.

### 2.5. Survey Administration

The survey was designed on Redcap^®^. The website was exclusively devoted to the survey, and the person had direct access to its information page. No incentives were offered for participation in this voluntary survey. There was no time limit for their completion.

### 2.6. Outcomes

Our principal endpoint was the percentage of departments reporting that they had a lactation room for pumping milk appropriate for nursing mothers and whether it met the criteria defined by the French Labor Code (article R.4152-13) [14]. It calls for a space separated from any other work space; ventilated and equipped with windows or other movable sashes opening directly to the outside; provided with a means of continuous air exchange; with appropriate lighting; with an adequate quantity of water or near a sink; with chairs appropriate for breastfeeding; kept constantly clean; and maintained at an appropriate temperature in hygienic conditions. The questionnaire studied all of these criteria.

The secondary outcome measures were the identification of different types of sites proposed as lactation spaces (dedicated room for lactation and breastfeeding, multiuse room prioritized for lactation, or an alternative space), the possibility of adapting the worker’s hours, access to the equipment necessary to express and store breast milk, the existence of a written policy concerning the continuation of breastfeeding at the workplace, the measures of support promoting continued breastfeeding (supervision, training, qualified resource persons, etc.), and setting up an educational program around breastfeeding.

### 2.7. Statistical Analysis

We analyzed only those questionnaires that allowed us to determine the principal endpoint. In view of the number of individuals, the analyses concerned the overall sample: the entire University Hospital as well as the RSPA obstetrics and pediatrics departments. The responses are reported as percentages of all respondents. For the secondary analyses, we described the spaces used by the departments and compared them according to both the presence of a lactation room (regardless of whether it met legal requirements or not) and the type of department. The responses were subsequently compared according to the presence of a lactation room, that is, for expressing/pumping milk (yes/no), and if yes, the type of room for milk expression (dedicated lactation room for both breastfeeding and pumping milk, a multi-use room prioritized for use for lactation, or an alternative space) and the type of department (perinatal health units, other hospital health care units or non-health care hospital units). If a department had several rooms for lactation, we used the best type of room to characterize it.

The chi-square test (or Fisher’s exact test, as appropriate) was used to compare the categorical (qualitative) variables. The threshold of significance was set at *p* < 0.05. The statistical analysis was conducted with SAS software (Statistics Program for Public Health on IBM-compatible Microcomputer, version 9.4, New York, NY, USA).

### 2.8. Ethical Approval

The relevant ethics committee (CPP Sud Est VI) approved this study on 20 October 2020 (N° 2020 CE 76). The study was reported to the Clermont-Ferrand University Hospital Data Protection Officer and is recorded on the hospital register as no. M201104. It has also been registered by the medical practice assessment commission of the Clermont-Ferrand University Hospital, which supervises the medical practice assessments on behalf of the quality assurance office and the hospital medical committee.

## 3. Results

### 3.1. Participation Rate

**Figure 1:**
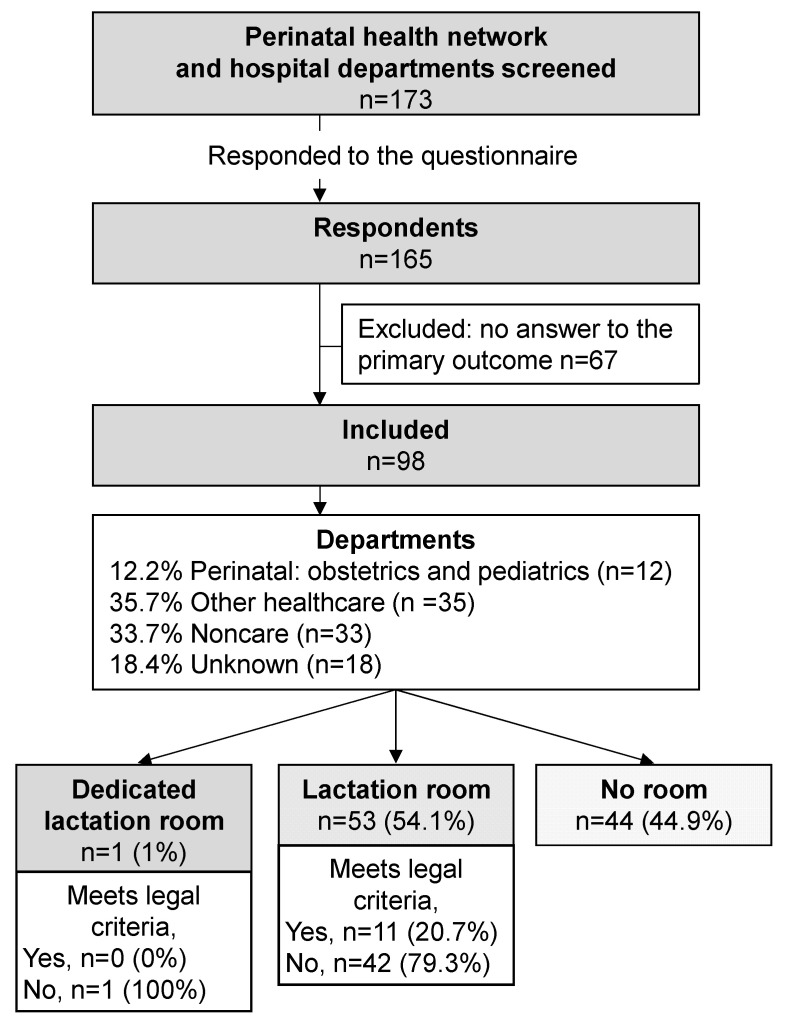
Flowchart.

Of the 173 eligible departments, we obtained responses from 165 for a participation rate of 95.4%. After the exclusion of 67 questionnaires that were incomplete, our study analyzed 98 questionnaires (Figure 1).

### 3.2. Baseline Characteristics of Participants and Departments

The respondents were principally healthcare managers or referral care professionals or midwife coordinators. More than half the responding departments provided patient care, and 12 of them were obstetrics and pediatrics departments. Nearly all the departments had had pregnant employees over the past 5 years. The respondents were in large majority women living with a partner with at least one child (Table 1). We did not find any statistically significant difference for the presence of a lactation room according to the characteristics of the individual respondents or of the departments.

### 3.3. Lactation Rooms

**Figure 2:**
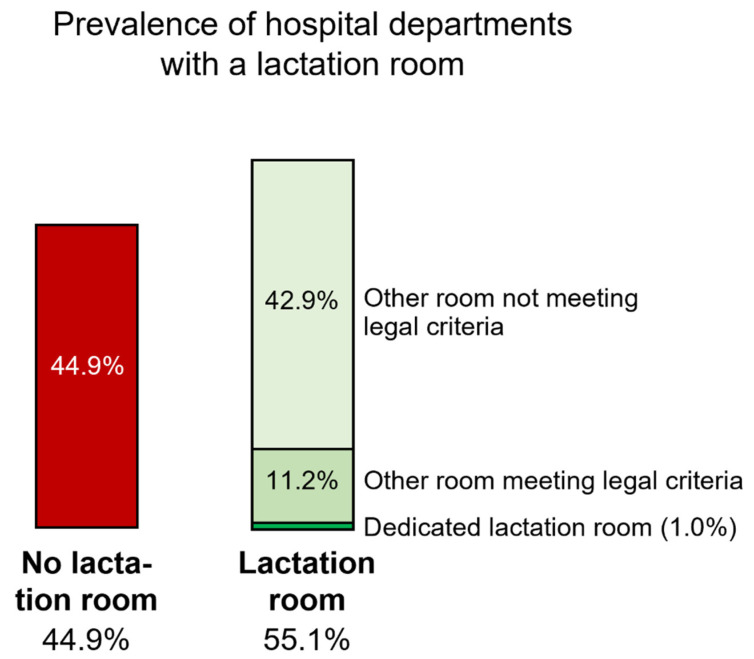
Prevalence of hospital departments with a lactation room.

Slightly more than half the departments (*n* = 54/98, 55.1%) offered a lactation room, that is, a room for pumping milk for its employees; only one room was dedicated to lactation and also intended for breastfeeding the baby in person (Figure 1). Among all of these spaces, only 11/54 (20.4%) met the legal requirements, that is, 11.2% of the responding departments (Figure 2). Appendix A (Table A1) details the rooms’ equipment and furnishings. Six departments offered more than one type of spaces for a total of 60 lactation rooms in 54 departments.

### 3.4. Equipment, Organization, and Possible Resources for Pumping Breast Milk

All these results are described in Table 2. Globally, few departments made a breast pump available to their employees. To store the milk, departments with a lactation room offered a refrigerator (dedicated or not) more often than departments without one (*p* = 0.01). Similarly, the departments with lactation rooms appeared to offer access to break time for pumping milk more frequently than the departments without one (*p* < 0.0001). Despite the presence of a daycare center at only one of the three university hospital sites, nine departments authorized their employees to go breastfeed their children there during work hours. One department authorized children to enter the department for feedings. Seven departments reported they have a policy concerning breastfeeding at return to work. One-third of the departments with at least one lactation room had identified a qualified resource person compared with only 2.9% of the other departments (*p* = 0.002).

### 3.5. Perinatal Healthcare Departments Compared with Other Healthcare Departments and with Noncare Departments

All of these analyses are presented in the Tables in the Appendix A (Table A2 and Table A3). Among them, we found numerous statistically significant differences between the perinatal departments (obstetrics and pediatrics) and the others (departments providing patient care as well as those that do not). That is, all perinatal departments offered a lactation room compared with 42.9% of the other patient care departments and 57.6% of the noncare departments (*p* = 0.001). Similarly, the perinatal departments often provided a breast pump, while none of the other departments did (*p* < 0.0001). Also similarly, the perinatal departments more often provided a refrigerator exclusively for milk storage or a shared department refrigerator rather than requiring women to bring in their personal coolers (*p* < 0.0001). Most perinatal departments (83.3%) had identified resource persons, unlike the other departments (other patient care departments: 5.7%; other noncare departments: 9.1%, *p* < 0.0001). Finally, 41.7% of the perinatal departments offered at least one form of breastfeeding education compared with 11.4% of the other care departments and 9.1% of the noncare departments (*p* = 0.02).

## 4. Discussion

Our cross-sectional survey covered a population of more than 9000 hospital-based workers and shows that only half of the responding departments in a university hospital and the perinatal departments in the region offered their employees a lactation room. Moreover, only 11.2% of these departments had a lactation room that met the requirements of the French regulations. Providing private places for milk expression appears to affect breastfeeding outcomes positively, although some of the studies in which it was the only support for breastfeeding failed to establish this result [16]. Moreover, the entire work environment plays a role in breastfeeding, that is, the existence of a lactation space, breastmilk pumping breaks, and organizational policies [9]. Despite the existence of a French law intended to protect women’s rights at workplace, we observed a gap between the statute’s mandates and real life at work [17]. Thus, the promotion of breastfeeding must be considered to be a collective societal responsibility [6].

### 4.1. Work Environment and Lactation Room

Dispositions present at women’s return to work can promote the continuation of breastfeeding in good conditions. A recent systematic review pointed out that these interventions activate underlying mechanisms by increasing awareness of working mothers, changing the workplace culture, fostering support from supervisors and co-workers, and providing enough time and adequate space and facilities for women to breastfeed or express breast milk during the workday [18]. Higher-quality lactation rooms were associated in a Dutch study with increased levels of satisfaction with them, perceived ease of milk expression at work, and perceived support from supervisors and co-workers for expressing milk in the workplace [19]. Our study identified only one lactation room also intended for direct breastfeeding, and very few departments had rooms meeting the legal requirements. A Spanish study of women working at a university also found a low score for the item “I can easily find a quiet place other than the bathroom at work to pump breast milk” on the Workplace Breastfeeding Support Scale questionnaire [20]. Our study found an association between the presence of a lactation room and some provisions and equipment, such as the availability of break time, the presence of a refrigerator for milk storage, and the identification of resource people for breastfeeding. Our secondary analyses also showed dispositions more favorable to breastfeeding in the perinatal departments, where, for example, all departments had a lactation room. This point may be explained by these departments’ greater awareness of the importance of breastfeeding and their employees’ greater alertness to their rights and their easier acceptance of these breaks for milk expression—all also facilitated by its strong acceptance by their colleagues. A U.S. study found that hospitals were more likely to offer lactation rooms than nonhospital organization [21]. The WHO and UNICEF recently reaffirmed the primordial role of midwives and nurses in breastfeeding support [22]. It is essential for hospitals to support breastfeeding initiation and duration in good conditions. Only in this way can healthcare professionals act as “breastfeeding promoters” to reduce the abandonment occurring at the first difficulties [23]—the most important of which is often return to work [6,7].

### 4.2. Break Time

Beyond a dedicated location, the ability to take break time is crucial for women who want to express their milk at their workplace, as numerous studies have reported. A recent American survey of 844 physicians found that one-third to one-half of them lack the time for breast pumping [24]. In the USA, despite the implementation of the Affordable Care Act in 2010, one study reported that 36.5% of respondents found that their work schedule was too demanding to take breaks for breastfeeding [25].

### 4.3. Support Measures

Access to resource persons and support by peers who have had positive experiences with breastfeeding have proven effective in prolonging breastfeeding duration [26,27]. Within hospitals, the perinatal departments can be identified as leaders in supporting nursing mothers. Nonetheless, in our study, only one-third of the departments knew about the existence of resource persons within their own hospital. Perinatal departments also have lactation consultants, whose support is known to produce favorable results on the initiation and duration of breastfeeding [28].

### 4.4. Equality of Access to These Dispositions

Among the strengths of this project, we must underline that our study is the first to look at the conditions allowing the continuation of breastfeeding on return to work in France. Nonetheless, our breastfeeding rate was low, and only one-third of the nursing mothers chose to continue breastfeeding on their return to work [28].

Numerous studies show inequalities between women for the continuation of breastfeeding on return to work. One study showed that manual workers and “intermediate employees” (i.e., those in subordinate positions) were less likely to be able to continue [29], while another found an important difference between hospitals, which were significantly more favorable to continued breastfeeding than nonhospital organizations [21]. Our study also showed an approach globally more favorable to continued breastfeeding in obstetric and pediatric departments than in other hospital departments. This point is also made in an American survey of emergency departments: one pediatric resident felt comfortable pumping her milk in other pediatric rotations because there were other residents who did; she was uncomfortable in the emergency department, where she had never seen anyone do it [30].

### 4.5. Limitations

One of the limitations of our study is the participation rate after we excluded the incomplete questionnaires of 56.6% of the departments; the study therefore does not represent our entire target population. Nonetheless, 98 respondents are a substantial number compared with numerous studies on the subject, especially in that we wrote to the supervisors of departments employing numerous women (both service and clerical workers) who might breastfeed; they reported their departmental policies and dispositions. Thus 98 respondents described availability for many more women. On this subject, we found several studies reporting surveys including fewer individuals—as few as 53 women [31] or even 37 [32]. Even a survey at the WHO included only 32 women [33]. This topic thus appears to be studied with fewer subjects than other surveys on other themes. Moreover, we did not find in the literature similar work aimed at identifying employers’ policies and actions promoting continued breastfeeding. The respondents in our study were mostly women and managers. Even though the population of nurses is predominantly female, one can wonder whether women did not feel more personally concerned by this study and were for that reason more likely to respond to the questionnaire. Our results did not find any difference between men and women respondents about the presence of a lactation room. Nonetheless, our population seems representative of health care workers, who are principally women [34].

## 5. Conclusions

This study documents the gap between the practices observed and those mandated by law concerning women’s breastfeeding at the workplace. It should call the attention of professionals with managerial responsibilities within departments or upper management to the need to implement improvements to facilitate women’s breastfeeding in the workplace. Our study found that only half the responding departments offered a lactation room, and only about 20% of those met the legal criteria for such a room. The practical implementation of programs to promote continued breastfeeding in the workplace could have a favorable effect on French rates of its initiation and continuation. In our study, almost half the departments surveyed did not offer any lactation room. Many barriers exist to the continuation of breastfeeding, including inconsistency in policies and the failure to enforce them in different countries [35]. Concrete measures—lactation rooms, break time, and institutional policy—must be envisioned to facilitate the pumping of breast milk on the return to work of hospital employees after maternity leave.

## Figures and Tables

**Table 1:** Characteristics of respondents and their departments according to the presence and type of lactation room.

	Departments with a Lactation Room				Departments without a Room	Room vs. No Room *p*-Value
	Total	Dedicated Room	Multiuse Room	Alternative Space		
	*n* = 54	*n* = 1	*n* = 15	*n* = 38	*n* = 44	
**Profession**	***n* = 46**	***n* = 1**	***n* = 13**	***n* = 32**	***n* = 34**	
Healthcare managers	25 (54.3)	0	8 (61.5)	17 (53.1)	21 (61.8)	0.12
Midwife coordinator	8 (17.4)	1 (100)	3 (23.1)	4 (12.5)	0	
Physician	1 (2.2)	0	0	1 (3.1)	1 (2.9)	
Director	1 (2.2)	0	0	1 (3.1)	2 (5.9)	
Referral healthcare worker	8 (17.4)	0	2 (15.4)	6 (18.7)	7 (20.6)	
Other	3 (6.5)	0	0	3 (9.4)	3 (8.8)	
**Description of department**	***n* = 46**	***n* = 1**	***n* = 13**	***n* = 32**	***n* = 34**	
**Healthcare department**	27 (58.7)	1 (100)	9 (69.2)	17 (53.1)	20 (58.8)	1.0
If yes, specialty						
Medicine	7 (15.2)	1 (100)	1 (7.7)	5 (15.6)	11 (32.3)	0.10
Surgery	7 (15.2)	0	2 (15.4)	5 (15.6)	6 (17.6)	0.77
Emergency, intensive care	4 (8.7)	0	0	4 (12.5)	4 (11.8)	0.72
Perinatal period ^1^	12 (26.1)	1 (100)	6 (40.0)	6 (15.8)	0	-
Pediatrics	5 (10.9)	0	3 (23.1)	2 (6.2)	0	-
Obstetrics	8 (17.4)	1 (100)	3 (23.1)	4 (12.5)	0	-
**Activity**	***n* = 54**	***n* = 1**	***n* = 13**	***n* = 32**	***n* = 44**	
Consultations	13 (24.1)	1 (100)	3 (20.0)	9 (23.7)	5 (11.4)	0.12
Operating suite	7 (13.0)	0	2 (13.3)	5 (13.2)	1 (2.3)	0.07
Day hospital	5 (9.3)	0	1 (6.7)	4 (10.5)	5 (17.2)	0.72
Inpatient hospitalization	20 (37.0)	0	7 (46.7)	13 (34.2)	17 (38.6)	0.87
**Risks and constraints**	***n* = 54**	***n* = 1**	***n* = 13**	***n* = 32**	***n* = 44**	
If yes,	36 (66.7)	1 (100)	10 (66.7)	25 (65.8)	25 (56.8)	0.32
Infectious	17 (31.5)	0	5 (33.3)	12 (31.6)	11 (25.0)	0.48
Toxic	8 (14.8)	0	3 (20.0)	5 (13.2)	6 (13.6)	0.87
Radiologic	9 (16.7)	0	2 (13.3)	7 (18.4)	4 (9.1)	0.37
Heat	2 (3.7)	0	1 (6.7)	1 (2.6)	1 (2.3)	1.0
Other	7 (13.0)	1 (100)	1 (6.7)	5 (13.2)	4 (9.1)	0.75
**Pregnancies and working conditions**	***n* = 46**	***n* = 1**	***n* = 13**	***n* = 32**	***n* = 34**	
Pregnancies of employees (past 5 years)	44 (95.6)	0	13 (100)	31 (96.9)	30 (88.2)	0.39
If yes,						
Switched to part-time/maternity leave	44 (100)	0	13 (100)	31 (100)	28 (93.3)	0.16
Medical visit at return from maternity leave						
Yes, all employees	28 (63.6)	0	8 (61.5)	20 (64.5)	19 (63.3)	1.0
Yes, some employees	8 (18.2)	0	4 (30.8)	4 (12.9)	5 (13.7)	
None	1 (2.3)	0	0	1 (3.2)	1 (3.3)	
I don’t know	7 (15.9)	0	1 (7.7)	6 (19.3)	5 (16.7)	
**Sociodemographic**	***n* = 45**	***n* = 1**	***n* = 12**	***n* = 32**	***n* = 34**	
**Age**						
20–29 years	0	-	-	-	3 (8.8)	0.11
30–39 years	16 (35.6)	0	3 (25.0)	13 (40.6)	8 (23.5)	
40–49 years	20 (44.4)	0	8 (66.7)	12 (37.5)	11 (32.3)	
≥50 years	9 (20.0)	1 (100)	1 (8.3)	7 (21.9)	12 (35.3)	
Sex						
Female	38 (84.4)	1 (100)	11 (91.7)	26 (81.2)	33 (97.1)	0.13
**Family situation**						
Lives with partner	39 (86.7)	1 (100)	10 (83.3)	28 (87.5)	28 (82.3)	0.6
Has at least one child	38 (84.4)	1 (100)	11 (91.7)	26 (81.2)	29 (85.3)	1.0
**Smoking: yes**	5 (11.6)	0	0	5 (15.6)	3 (34.0)	1.0

^1^ Perinatal period = obstetrics + pediatrics. One person leads both an obstetrics and a pediatrics department. Values are presented as N (%) Dedicated lactation room for breastfeeding/lactation (single user or partitioned for multiple user); multi-use room: a nondedicated space, such as a meeting room, wellness room, vacant or rarely used office, or other space that is prioritized for use by nursing mothers each time they have a need to express milk; alternative space: when every space has been examined, and there are no apparent options for providing or creating a room with a door: flexibility and creativity are key.

**Table 2:** Equipment, organization, and possible resources for pumping/expressing breast milk.

	Departments with a Lactation Room				Departments without a Room	Room vs. No Room *p*-Value
	Total	Dedicated Room	Multiuse Room	Alternative Space		
	*n* = 54	*n* = 1	*n* = 15	*n* = 38	*n* = 44	
**Material for collecting milk**						
Breast pump in the room	1 (1.8)	0	0	1 (2.6)	0	-
Breast pump if women forgot theirs	5 (9.3)	0	2 (13.3)	3 (7.9)	1 (2.3)	
Professional breast pump	48 (88.9)	1 (100)	13 (86.7)	34 (89.5)	43 (97.7)	
**Storage of breast milk**						
Dedicated refrigerator	7 (13.0)	0	3 (20.0)	4 (10.5)	0	0.010
Shared refrigerator	43 (79.6)	0	12 (80.0)	31 (81.6)	35 (79.5)	
Personal cooler	4 (7.4)	1 (100)	0	3 (7.9)	9 (20.4)	
**Possibility of break time**	*n* = 48	*n* = 1	*n* = 14	*n* = 33	*n* = 38	
All professionals	37 (77.1)	1 (100)	11 (78.6)	25 (75.8)	10 (26.3)	
≥50% of professionals	2 (4.2)	0	0	2 (6.1)	0	<0.001
<50% of professionals	3 (6.2)	0	1 (7.1)	2 (6.1)	4 (10.5)	
No	6 (12.5)	0	2 (14.3)	4 (12.1)	24 (63.2)	
**Distribution of break time**	*n* = 54	*n* =1	*n* = 15	*n* = 38	*n* = 44	
Agreement between employee/employer	13 (24.1)	0	4 (26.7)	9 (23.7)	2 (4.5)	0.010
Depending on how busy the department is	31 (57.4)	0	8 (53.3)	23 (60.5)	9 (20.4)	<0.001
Favor meal time and regular break time	12 (22.2)	1 (100)	0	11 (28.9)	5 (11.4)	0.20
**Possibility of continuing to work while pumping milk**	2 (3.7)	0	1 (6.7)	1 (2.6)	1 (2.3)	1.0
**Facility’s policy about breastfeeding**	4/48 (8.3)	0	2/14 (14.3)	2/33 (6.1)	3/38 (7.9)	1.0
**Support measures**	*n* = 48	*n* = 1	*n* = 14	*n* = 33	*n* = 35	
Qualified resource persons						
Yes	16 (33.3)	1 (100)	6 (42.9)	9 (27.3)	1 (2.9)	0.002
Don’t know	19 (39.6)	0	6 (42.9)	13 (39.4)	21 (60.0)	
**Educational measures offered**	*n* = 54	*n* = 1	*n* = 15	*n* = 38	*n* = 44	
At least one of these measures	21 (38.9)	1 (100)	4 (26.7)	8 (21.1)	7 (15.9)	0.25
Educational resources	2 (9.5)	1 (100)	0	1 (12.5)	0	
Information within the facility	6 (28.6)	1 (100)	0	5 (62.5)	1 (14.3)	
Promotion of breastfeeding	5 (23.8)	1 (100)	1 (25.0)	3 (37.5)	1 (14.3)	
Census of professionals who have already breastfed in the departments	2 (9.5)	0	0	2 (25.0)	0	
Referral to associations	3 (14.3)	0	1 (25.0)	2 (25.0)	0	
Encouraging professionals to take childbirth classes	7 (33.3)	1 (100)	2 (50.0))	3 (37.5)	1 (14.3)	
Informing the professionals of their rights	7 (33.3)	0	1 (25.0)	5 (62.5)	1 (14.3)	
Other	1 (4.8)	0	1 (25.0)	0	5 (71.4)	

Values are presented as N (%) or n/N (%) Dedicated lactation room for breastfeeding/lactation (single user or partitioned for multiple user); multi-use room: a nondedicated space, such as a meeting room, wellness room, vacant or rarely used office, or other space that is prioritized for use by nursing mothers each time they have a need to express milk; alternative space: when every space has been examined, and there are no apparent options for providing or creating a room with a door: flexibility and creativity are key.

## Data Availability

Not applicable.

## Supplementary Materials

The following supporting information can be downloaded at: https://www.mdpi.com/article/10.3390/nu14173463/s1, Questionnaire S1: Workplace lactation support study questionnaire (French).

## Appendix A

**Table A1:** Characteristics of lactation rooms.

Characteristics of Rooms	Dedicated Room *n* = 1	Multiuse Room *n* = 15	Alternative Space *n* = 44
**Equipment and furnishings**			
Electric outlet	1 (100)	15 (100)	42 (95.5)
Lock	1 (100)	6 (40.0)	23 (52.3)
Ventilation and window	1 (100)	11 (73.3)	31 (70.5)
Continuous air renewal	0	9 (60.0)	18 (40.9)
Lighting	1 (100)	15 (100)	44 (100)
Clean water source	0	10 (66.7)	29 (65.9)
Table	1 (100)	14 (93.3)	7 (15.9)
**Comfort**			
Chair	1 (100)	9 (60.0)	28 (63.7)
Appropriate temperature	1 (100)	14 (93.3)	37 (84.1)
**Cleanliness**			
Cleaned daily	1 (100)	14 (93.3)	37 (84.1)
**Meets legal requirements**	0	3 (20.0)	8 (18.2)

**Table A2:** Baseline characteristics according to type of department.

	Departments			*p*-Value
	Perinatal *n* = 12	Other Healthcare *n* = 35	Noncare *n* = 33	
**Pregnancies and working conditions**				
Pregnancies of employees (past 5 years)	11 (91.7)	35 (100)	28 (84.8)	-
If yes,				
Switched to part-time/maternity leave	11 (100)	35 (100)	26 (92.9)	0.27
Medical visit at return from maternity leave				
Yes, all employees	6 (54.5)	24 (68.6)	17 (60.7)	0.93
Yes, some employees	3 (27.3)	5 (14.3)	5 (17.9)	
None	0	1 (2.9)	1 (3.6)	
I don’t know	2 (18.2)	5 (14.3)	5 (17.9)	
**Sociodemographic**	***n* = 11**	***n* = 35**	***n* = 33**	
**Age**				
20 years	0	0	3 (9.1)	0.002
30–39 years	4 (36.4)	4 (11.4)	16 (48.5)	
40–49 years	6 (54.5)	19 (54.3)	6 (18.2)	
≥50 years	1 (9.1)	12 (34.3)	8 (24.2)	
**Sex**				
Female	11 (100)	30 (85.7)	30 (90.9)	0.54
**Family situation**				
Lives with partner	9 (81.8)	27 (77.1)	31 (93.9)	0.12
Has at least one child	10 (90.9)	32 (91.4)	25 (75.8)	0.19
**Smoker**: Yes	0	5 (14.3)	3 (9.4)	0.67

Values are presented as N (%) or n/N (%). Dedicated lactation room for breastfeeding/lactation (single user or partitioned for multiple user); multi-use room: a nondedicated space, such as a meeting room, wellness room, vacant or rarely used office, or other space that is prioritized for use by nursing mothers each time they have a need to express milk; alternative space: when every space has been examined, and there are no apparent options for providing or creating a room with a door: flexibility and creativity are key.

**Table A3:** Equipment, organization, and possible resources for pumping breast milk by type of department.

	Departments			*p*-Value
	Perinatal *n* = 12	Other Healthcare *n* = 35	Noncare *n* = 33	
**Lactation room**	12 (100)	15 (42.9)	19 (57.6)	0.001
If yes, what type				
Dedicated room	1 (8.3)	0	0	
Multi-use room prioritized for lactation	6 (50.0)	3 (20.0)	4 (21.0)	0.090
Alternative space	5 (41.7)	12 (80.0)	15 (78.9)	
**Material for collecting milk**				
Breast pump belonging to department	1 (8.3)	0	0	<0.001
Breast pump if a woman has forgotten hers	5 (41.7)	0	0	
Professional breast pump	6 (50.0)	35 (100)	33 (100)	
**Storage**				
Refrigerator dedicated to storage of breast milk	6 (50.0)	1 (2.9)	0	<0.001
Refrigerator shared by all departmental staff	5 (41.7)	33 (94.3)	25 (75.8)	
Personal cooler	1 (8.3)	1 (2.9)	8 (24.2)	
**Possibility of break time**				
All professionals	9 (75.0)	17 (48.6)	18 (54.5)	
Some	1 (8.3)	2 (5.7)	6 (18.2)	0.18
None	2 (16.7)	16 (45.7)	9 (27.3)	
If yes, distribution of break time				
Agreement between employee/employer	2 (16.7)	2 (5.7)	11 (33.3)	0.010
Depending on how busy the department is	8 (66.7)	14 (40.0)	16 (48.5)	0.32
Favor meal time and regular break time	1 (8.3)	7 (20.0)	8 (24.2)	0.60
**Possibility of continuing to work while pumping milk**	0	2 (5.7)	1 (3.0)	-
**Facility’s policy about breastfeeding**	0	2 (5.7)	5 (15.1)	0.30
**Support measures**				
Resource persons qualified				
Yes	10 (83.3)	2 (5.7)	3 (9.1)	<0.001
Don’t know	0	20 (57.1)	20 (60.6)	
**Education measures offered**				
At least one of these measures	5 (41.7)	4 (11.4)	3 (9.1)	0.020

Values are presented as N (%) or n/N (%). Dedicated lactation room for breastfeeding/lactation (single user or partitioned for multiple user); multi-use room: a nondedicated space, such as a meeting room, wellness room, vacant or rarely used office, or other space that is prioritized for use by nursing mothers each time they have a need to express milk; alternative space: when every space has been examined, and there are no apparent options for providing or creating a room with a door: flexibility and creativity are key.
