# Supplementary material for: Workplace Lactation Support: A Cross-Sectional Study in a University Hospital and a Perinatal Network
Source: Nutrients. 2022 Aug 24;14(17):3463. doi: 10.3390/nu14173463 (PMC9460242; doi:10.3390/nu14173463)
Supplement: Supplementary file 1 [file nutrients-14-03463-s001.zip › nutrients-1839680-supplementary.pdf]

Allaitement maternel et reprise du travail

Madame, Monsieur,

Vous avez été invité à participer à une étude appelée "Allaitement maternel et reprise du travail : Enquête déclarative sur les aménagements disponibles dans les services hospitaliers".

Cette étude s'intéresse aux dispositions mises en place afin de faciliter la poursuite de l'allaitement maternel à la reprise du travail dans les services hospitaliers. Elle s'inscrit dans le cadre d'un travail de recherche de fin d'études d'une étudiante sage-femme, Marina Stankovic. Cette étude est sous la responsabilité de Chloé Barasinski, sage-femme de recherche au sein de la maternité du CHU de Clermont-Ferrand.
La réalisation du mémoire est faite en collaboration avec : l'équipe de recherche Institut Pascal, Axe TGI-PEPRADE, CNRS-UMR 6602, le réseau de santé périnatal d'Auvergne, le service de Santé au travail du CHU de Clermont-Ferrand, l'école de sage-femme ; et avec le soutien de la direction des ressources humaines du CHU de Clermont-Ferrand.

1. Pourquoi cette étude

L'allaitement maternel est recommandée de manière exclusive pendant 6 mois et de poursuivre l'allaitement au sein jusqu'à l'âge de 2 ans ou plus. A la reprise du travail, les mères qui allaitent encore ont souvent besoin d'exprimer leur lait sur leur lieu de travail. Cette étude vise faire un état des lieux des services de santé bénéficiant d'un local adéquat afin que les professionnels puissent recueillir leur lait sur le lieu de travail et de décrire les autres dispositions mises en place (local, matériel, stockage, temps de pause, politique de l'établissement, mesures de soutien) afin de faciliter la poursuite de l'allaitement à la reprise du travail..

2. L'étude en pratique

Cette étude consiste à interroger l'ensemble des personnes en charge de l'encadrement d'un service du CHU de Clermont-Ferrand (quel que soit le statut de cette personne) ou d'un service de maternité du Réseau de Santé Périnatale d'Auvergne via un questionnaire anonyme. Ce questionnaire est accessible sur la plateforme RedCap.
Votre participation à cette étude se termine à la fin du remplissage du questionnaire.

3. Confidentialité et sécurité des données

Cette étude a reçu un avis favorable du Comité de Protection des Personnes Sud Est VI le 20/10/2020 n°: 2020 / CE 76. Aucune donnée nominative ou identifiante n'est relevée dans l'étude. Le personnel impliqué dans l'étude est soumis au secret professionnel. Conformément aux dispositions du Règlement Européen de Protection des Données personnelles (RGPD) entré en vigueur le 25 mai 2018 et de la loi Informatique et Libertés du 6 janvier 1978 modifiée (par la loi n° 2018-493 du 20 juin 2018), vous disposez d'un droit d'accès et de rectification.

Cette étude a fait l'objet d'une déclaration auprès du DPD (Délégué à la protection des données) du CHU de Clermont-Ferrand.

Vos droits

Votre participation à cette étude est entièrement libre et volontaire. Vous êtes libre de refuser d'y participer ainsi que de mettre un terme à votre participation à n'importe quel moment, sans encourir aucune responsabilité ni aucun préjudice de ce fait (aucune modification de prise en charge).
Conformément au RGPD et à la loi Informatique et Libertés du 6 janvier 1978 modifiée (par la loi n° 2018-493 du 20 juin 2018), vous avez le droit d'avoir communication des données vous concernant et le droit de demander éventuellement l'effacement de ces données si vous décider d'arrêter votre participation à l'étude. Vous avez également la possibilité de vérifier l'exactitude des informations que vous aurez fournies et la possibilité de demander éventuellement leur correction. Vos données seront conservées 2 ans après la fin de l'étude. Ces droits pourront s'exercer à tout moment en adressant une demande écrite à Chloé Barasinski, cbarasinski@chu-clermontferrand.fr, sage-femme au sein de la maternité du CHU de Clermont-Ferrand.

4. Obtention d'informations complémentaires :
Si vous le souhaitez, vous pouvez durant toute la durée de l'étude contacter le responsable principal pour obtenir des précisions ou des informations complémentaires : Chloé Barasinski.

Pour toute question relative la protection de vos données personnelles : vous pouvez contacter le délégué à la protection des données, Michel Rubio, dpd@chu-clermontferrand.fr CHU DQGRDU 58 rue Montalembert 63003 Clermont-Ferrand

La base légale de ce traitement est l'intérêt légitime du CHU de Clermont-Ferrand, en tant qu'établissement de santé Hospitalo-Universitaire à vocation de recherche, à mettre en œuvre le traitement de données médicales à des fins de recherche scientifique (article 9.2 du RGPD).

En vous remerciant par avance de vos réponses, de votre collaboration et du temps que vous mettez à contribution pour la réalisation de ce projet.

Très cordialement,

Marina Stankovic (étudiante sage-femme),
Chloé Barasinski (Sage-femme de recherche clinique et directrice du mémoire),
Pr Frédéric Dutheil (Responsable de service de Santé au Travail du CHU de Clermont-Ferrand),
Pr Françoise Vendittelli (Présidente du Réseau de Santé Périnatal d'Auvergne).

AMENAGEMENT DES LOCAUX

I - Locaux

Accessible à l'ensemble des professionnelles travaillant dans le service ; toute femme, exerçant dans le service, tous postes confondus, et souhaitant allaiter ou exprimer son lait pourra se rendre dans un lieu à disposition.

a. Au sein de votre service, disposez-vous ou avez-vous accès à un espace non réservé utilisé en priorité par les mères qui allaitent dès qu'elles ont besoin d'exprimer leur lait (comme une salle de réunion, une salle de bien-être, un bureau vacant ou rarement utilisé, ou tout autre espace utilisé) ?	◯ Oui<br>◯ Non
Cet espace se situe	◯ Au sein même du service<br>◯ Dans un service à proximité immédiate (5 minutes ou moins)<br>◯ Dans un service plus éloigné (plus de 5 minutes)
Comprend-t-il en terme d'équipement et de mobilier :	☐ Une prise électrique<br>☐ Un verrou pour fermer la porte et une politique bien communiquée qui garantit qu'il n'y aura pas d'intrusion<br>☐ Une aération et espace muni d'une fenêtre ou autres ouvrants à châssis mobile donnant directement sur l'extérieur<br>☐ Un mode de renouvellement d'air continu<br>☐ Un éclairage convenable<br>☐ Une source d'eau propre à proximité<br>☐ Une table ou surface plane pour le tire-lait et l'équipement<br>☐ Autre(s) (précisez)
Si autre, précisez:	________________________________
En terme de confort :	☐ Une chaise confortable<br>☐ Une température convenable<br>☐ Autre(s) (précisez)
Si autre, précisez:	________________________________
En terme de propreté	☐ Un état de propreté constant (nettoyage quotidien)<br>☐ Autre(s) (précisez)

Si autre précisez:	__________________________
b. Au sein de votre service, disposez-vous ou avez-vous accès à un espace alternatif ? (espace n'étant pas dédié en priorité pour les mères souhaitant exprimer leur lait)	◯ Oui<br>◯ Non
Cet espace se situe:	◯ Au sein même du service<br>◯ Dans un service à proximité immédiate (5 minutes ou moins)<br>◯ Dans un service plus éloigné (plus de 5 minutes)
Quels formes peut-il prendre?	☐ Cabine à paroi haute avec un rideau ou une porte<br>☐ Coin séparé par un rideau ou cloisonné d'une pièce plus grande<br>☐ Bureau du cadre ou autre bureau qui peut être libéré pour l'usage de la mère<br>☐ Vestiaire<br>☐ Chambre ou salle d'examen vide au sein du service<br>☐ Chambre ou salle d'examen vide au sein d'un autre service<br>☐ Autre(s) (précisez)
Si autre précisez :	__________________________
Comprend-t-il en terme d'équipement et de mobilier :	☐ Une prise électrique<br>☐ Un verrou pour fermer la porte et une politique bien communiqué qui garantit qu'il n'y aura pas d'intrusion<br>☐ Une aération et espace muni d'une fenêtre ou autres ouvrants à châssis mobile donnant directement sur l'extérieur<br>☐ Un mode de renouvellement d'air continu<br>☐ Un éclairage convenable<br>☐ Une source d'eau propre à proximité<br>☐ Une table ou surface plane pour le tire-lait et l'équipement<br>☐ Autre(s) (précisez)
Si autre précisez:	__________________________
En terme de confort:	☐ Une chaise confortable<br>☐ Une température convenable<br>☐ Autre(s) (précisez)
Si autre, précisez:	__________________________
En terme de propreté:	☐ Un état de propreté constant (nettoyage quotidien)<br>☐ Autre(s) (précisez)
Si autre, précisez :	__________________________

c. Au sein de votre service, disposez-vous ou avez-vous accès à un espace dédié à la lactation c'est-à-dire une "salle d'allaitement" (monoposte ou cloisonnée pour plusieurs utilisatrices) ?	◯ Oui<br>◯ Non
Cet espace se situe:	◯ Au sein même du service<br>◯ Dans un service à proximité immédiate (5 minutes ou moins)<br>◯ Dans un service plus éloigné (plus de 5 minutes)
Comprend-t-il en terme d'équipements et de mobilier:	☐ Une prise électrique<br>☐ Un verrou pour fermer la porte<br>☐ Une aération et espace muni d'une fenêtre ou autres ouvrants à châssis mobile donnant directement sur l'extérieur<br>☐ Un mode de renouvellement d'air continu<br>☐ Un éclairage convenable<br>☐ Une source d'eau propre à proximité<br>☐ Un lavabo<br>☐ Du savon<br>☐ Une table ou surface plane pour le tire-lait et l'équipement<br>☐ Autre(s) (précisez)
Si autre précisez:	______________________________
En terme de confort:	☐ Une chaise confortable<br>☐ Une température convenable<br>☐ Un miroir<br>☐ D'étagères ou casiers pour stocker des fournitures personnelles et des fournitures d'allaitement.<br>☐ Autre(s) (précisez)
Si autre, précisez :	______________________________
En terme de propreté :	☐ Un état de propreté constant (nettoyage quotidien)<br>☐ Des lingettes désinfectantes<br>☐ L'équipement pour l'entretien du matériel (éponges, produit vaisselle, torchon)<br>☐ Autre(s) (précisez)
Si autre précisez :	______________________________
En terme de possibilité de travail :	☐ Poste de travail (ordinateur et un accès à Internet)<br>☐ Autre(s) (précisez)<br>☐ Pas de possibilité de travail
Si autre, précisez :	______________________________

Accessible pour une certaine catégorie de professionnelles exerçant dans le service (secrétariat, personne chargé d'encadrement, praticien, consultant, chercheur...)

a. Existe-t-il d'autres espace accessible que ceux cités précédemment pour certaines catégories de professionnelles?

- ◯ Oui
- ◯ Non

Si oui, precisez quelle(s) catégorie(s) professionnelle(s) ?

____

Si oui, sous quelle(s) forme(s) ?

- ☐ Bureau individualisé fermé
- ☐ Bureau partagé fermé
- ☐ Bureau partagé non fermé
- ☐ Salle de consultation
- ☐ Salle de réunion
- ☐ Autre(s) (précisez)

Si autre précisez :

____

II- Matériel pour le recueil du lait

1) Afin de recueillir leur lait, les professionnelles du service ont-elles accès (dans le service ou à proximité immédiate soit < 5 minutes)?

- ◯ A un tire-lait acheté ou loué par le service ou l'établissement, disponible dès qu'elles en ont besoin
- ◯ A un tire-lait acheté ou loué par le service ou l'établissement, en cas d'oubli de leur matériel, exceptionnellement
- ◯ Les professionnelles emmènent leur propre tire-lait

III- Stockage

1) Les professionnelles du service ont-elles accès à un espace de stockage du lait maternel (dans le service ou à proximité immédiate soit < 5 minutes)?

- ◯ Oui, dans un réfrigérateur dédié à la conservation du lait maternel (température conforme et contrôlé 0°C +4°C)
- ◯ Oui, dans un réfrigérateur commun aux membres du personnel du service (en salle de repos, température conforme 0°C +4°C)
- ◯ Non (la professionnelle conserve le lait dans sa glacière personnelle avec de la glace).

2) Les professionnelles du service ont-elles accès à un congélateur (afin de conserver les packs de glace notamment) :

- ◯ Oui
- ◯ Non

LES TEMPS DE PAUSE

1) Les professionnelles en poste dans le service ont-elles la possibilité de bénéficier d'un temps de pause afin d'exprimer leur lait, ou de se déplacer pour allaiter leur enfant, ou allaiter leur enfant directement dans leur service :	◯ Oui, l'ensemble des professionnelles (toutes catégories) peuvent y avoir accès<br>◯ Oui, mais seulement une partie des professionnelles peuvent y avoir accès (≥ 50% des catégories professionnelles)<br>◯ Oui, mais seulement la moitié ou moins des professionnelles du service peuvent y avoir accès (< 50% des différentes catégories professionnelles)<br>◯ Non, aucune des professionnelles du service ne peut bénéficier d'une pause pour exprimer son lait ou allaiter son enfant directement.
a) Si oui, comment les professionnelles peuvent-elles prendre et répartir le temps de pause pour exprimer leur lait au sein du service :<br>Concernant la possibilité de prendre ces 2 pauses de 30 minutes au cours de la journée :	◯ L'ensemble des professionnelles a facilement la possibilité de prendre ces 2 pauses de 30 minutes<br>◯ Une partie (≥ 50% des professionnelles) a facilement la possibilité de prendre ces 2 pauses de 30 minutes<br>◯ Une petite proportion (< 50% des professionnelles) a facilement la possibilité de prendre ces 2 pauses de 30 minutes
Concernant la répartition de ces 2 pauses de 30 minutes au cours de la journée :	☐ La période où le travail est suspendu pour l'expression du lait est déterminée en accord entre le salarié et l'employeur.<br>☐ En l'absence d'accord concernant la période où le travail est suspendu, cette dernière est placée au milieu de chaque demi-journée.<br>☐ Le temps de pause est réalisé en fonction de l'activité du service.<br>☐ La salariée est encouragée à utiliser ses heures habituelles de repas et de pause pour exprimer son lait<br>☐ Des dispositions sont prises pour que les salariés puissent continuer à travailler pendant l'expression du lait.
b. Si oui, existe-il des conditions particulières pour que les femmes aient la possibilité d'exprimer leur lait pendant le travail : les professionnelles doivent-elles joindre :	☐ Un certificat médical attestant que la femme allaite son enfant<br>☐ Une autorisation préalable par la médecine du travail<br>☐ Autre(s) (précisez)<br>☐ Il n'existe pas de conditions particulières
Si autres, précisez :	__________________________________

c.S'il existe une crèche au sein de la structure hospitalière ou si l'enfant est gardé à proximité du lieu de travail :	☐ Une certaine catégorie des professionnelles a la possibilité de se rendre à cette crèche ou sur le lieu de garde de son enfant pour l'allaiter.<br>☐ L'ensemble des professionnelles a la possibilité de se rendre à cette crèche ou sur le lieu de garde de son enfant pour l'allaiter.<br>☐ La personne qui s'occupe de l'enfant peut l'amener à la mère pendant sa pause pour l'allaiter directement au sein et ce, pour une certaine partie des femmes.<br>☐ La personne qui s'occupe de l'enfant peut l'amener à la mère pendant sa pause pour l'allaiter directement au sein et ce, pour l'ensemble des femmes.<br>☐ La professionnelle ne pourra se déplacer ni allaiter son enfant au sein du service mais pourra exprimer son lait.
Si seulement une partie des femmes peuvent aller allaiter leur enfant à la crèche ou l'allaiter directement dans le service, lesquelles ?	__________________________________

POLITIQUE DE L'ETABLISSEMENT DECLINEE DANS LE SERVICE

1) La structure bénéficie-t-elle d'une politique écrite / d'un règlement intérieur concernant la poursuite de l'allaitement maternel sur le lieu de travail ?

- ◯ Oui
- ◯ Non

a. Si oui, est-elle :

- ☐ Affichée au sein du service
- ☐ Communiquée aux professionnelles lorsqu'elles sont embauchées au sein de la structure hospitalière
- ☐ Diffusée aux professionnelles au cours de leur grossesse ou de leur retour de congés maternité
- ☐ Abordée avec les professionnelles souhaitant allaiter et souhaitant avoir des informations lorsqu'elles en font la démarche/demande
- ☐ Autre(s) (précisez)

Si autres, précisez: ______________________

MESURE DE SOUTIEN

1) La structure bénéficie d'options de transition de retour au travail ?	◯ Oui<br>◯ Non
a.Si oui:	☐ La professionnelle peut reprendre en alternant 1 journée de travail et 1 journée de repos (afin de faciliter son organisation entre travail et allaitement)<br>☐ Le retour progressif au travail est possible : l'employé peut reprendre progressivement son travail selon des horaires adaptés sur une période de quelques semaines.<br>☐ Des options de télétravail partiel ou total, travail à domicile sont disponibles.<br>☐ Des horaires à temps partiel après un congé maternité et/ou parental avec maintien des avantages sociaux, dans le même poste ou un poste similaire, sont disponibles<br>☐ La reprise du travail est possible dans un secteur où la professionnelle peut plus facilement concilier son travail et la poursuite de son allaitement (accès simplifié aux temps de pause, locaux disponibles à proximité, ...)<br>☐ Autre(s) (précisez)
Si autres, précisez:	__________________________________
2) Votre service ou votre établissement dispose-t-il de personnes qualifiées/ressources identifiées afin de soutenir les femmes au cours de l'allaitement (consultante en lactation, professionnels qualifiés...)?	◯ Oui<br>◯ Non<br>◯ Je ne sais pas
a. Si oui, combien à l'échelle de l'établissement? (ne pas répondre si vous ne savez pas)	__________________________________
b. Si oui, combien à l'échelle du service?	__________________________________

EDUCATION AUTOUR DE L'ALLAITEMENT

1) Parmi ces exemples de mesures d'éducation autour de l'allaitement, lesquelles sont pratiquées dans votre établissement (plusieurs réponses possibles) ?

- [ ] Fournir aux professionnelles des liens en ligne vers des ressources éducatives et/ou de la documentation sur la grossesse, l'allaitement maternel, et la reprise du travail
- [ ] Délivrer les informations à propos de l'allaitement et la reprise du travail au sein de votre structure (où, quand et comment tirer son lait sur son lieu de travail)
- [ ] Réaliser la promotion de l'allaitement maternel selon les recommandations en vigueur (l'OMS recommande l'allaitement exclusif au sein pendant les six premiers mois de la vie et il doit se poursuivre ensuite jusqu'à l'âge de deux ans au moins, en l'associant à une alimentation de complément qui convienne)
- [ ] Recenser les professionnelles ayant déjà allaité dans les services afin de leur adresser les employées en cours de grossesse qui souhaitent allaiter au travail
- [ ] Orienter les professionnelles vers des associations traitant de l'allaitement maternel et la reprise du travail
- [ ] Encourager les professionnelles à pratiquer des cours de préparation à la naissance et à la parentalité autour de l'allaitement maternel
- [ ] Informer les professionnelles de leurs droits en matière d'allaitement (durant la grossesse, lorsque les femmes sont embauchées ou à leur retour de congés maternité)
- [ ] Autre(s) (précisez)
- [ ] Aucune intervention particulière n'est prévue concernant l'allaitement

Si autre, précisez : ______________________________

DESCRIPTIF DE VOTRE SERVICE

1) Dans les 5 dernières années, une ou des femmes travaillant dans votre service ont-elles eu un enfant ?	◯ Oui<br>◯ Non
a. Si oui, parmi ces femmes, certaines ont-t-elles eu recours au temps partiel et/ou à un congé parental ?	◯ Oui<br>◯ Non
b. Si oui, les femmes ont-elles bénéficié d'une visite médicale au service de santé au travail à la fin de leur congé maternité ?	◯ Oui, toutes<br>◯ Oui, la grande majorité<br>◯ Oui, une partie seulement<br>◯ Non<br>◯ Je ne sais pas
2) Quelle(s) est, ou sont, la, ou les, filières professionnelles présentes dans votre service ?	☐ La filière soignante<br>☐ La filière de rééducation<br>☐ La filière médico-technique (préparateur en pharmacie, technicien de laboratoire<br>☐ La filière administrative<br>☐ La filière technique (ingénieur hospitalier...)<br>☐ La filière ouvrière<br>☐ La filière socio-éducative<br>☐ Médecins et/ou pharmaciens
3) Coordonnez-vous un service de soin?	◯ Oui<br>◯ Non
a. Si vous coordonnez un service de soins, dans quelle spécialité se situe-t-il ?	☐ Médecine<br>☐ Urgences, soins intensifs, réanimation<br>☐ Chirurgie<br>☐ Obstétrique<br>☐ Pédiatrie
Si Obstétrique, quel est le type de votre maternité?	◯ Type 1<br>◯ Type 2<br>◯ Type 3
b. Quelle activité est prévue dans votre service ?	☐ Consultations<br>☐ Hôpital de jour<br>☐ Hospitalisation complète<br>☐ Bloc opératoire<br>☐ Autre(s) (précisez)
Si autre, précisez:	______________________
c. Si vous êtes un service de soins, combien de lits d'hospitalisation comptent les secteurs sous votre responsabilité ?	______________________
3bis) Votre service est-il au sein du CHU de Clermont-Ferrand?	◯ Oui<br>◯ Non

4) Comment est organisé le temps de travail des personnels de votre service (organisation du temps de travail de 50% ou plus du personnel) ?	☐ Horaires de jour<br>☐ Horaires de nuit<br>☐ Alternance jour/nuit<br>☐ Spécifiez si besoin
Spécifiez si besoin:	__________________
5) Votre service comporte-t-il des risques ou contraintes :	☐ Infectieux (patientes porteurs d'infection pour plus de 50% d'entre eux)<br>☐ Toxiques (manipulation de produits pouvant être à risque dans le contexte de la périnatalité, par exemple cytostatiques, entonox à haute dose).<br>☐ Radiologiques<br>☐ Thermiques (par exemple travail en secteur chaud ou froid)<br>☐ Autre(s) (précisez)<br>☐ Le service ne comporte pas de risques ou contraintes
Si autres, précisez:	__________________

A PROPOS DE VOUS

1) Quelle est votre profession?

____

2) Quel est votre sexe?

- ◯ Homme
- ◯ Femme

3) Quelle est votre catégorie d'âge :

- ◯ 20-29 ans
- ◯ 30-39 ans
- ◯ 40-49 ans
- ◯ 50-59 ans
- ◯ 60 ans et plus

4) Quelle est votre situation familiale :

- ◯ vivant seule
- ◯ vivant en couple

5) Avez-vous des enfants ?

- ◯ Oui
- ◯ Non

Si oui, combien :

____

6) Quel est votre niveau de :
a. Stress au travail

aucun stress — stress maximal imaginable

(Place a mark on the scale above)

b. Stress à la maison

aucun stress — stress maximal imaginable

(Place a mark on the scale above)

c. Burn-out / épuisement :

aucun burn-out — burn-out maximal imaginable

(Place a mark on the scale above)

d. Latitude décisionnelle / autonomie au travail:

aucune autonomie — autonomie maximale imagineable

(Place a mark on the scale above)

e. Demande psychologique au travail (charge de travail) :

aucune demande — demande maximale imaginable

(Place a mark on the scale above)

f. Soutien de votre hiérarchie dans le cadre du travail :

aucun soutien — soutien maximal imaginable

(Place a mark on the scale above)

g. Soutien de vos collègues dans le cadre du travail:

aucun soutien — soutien maximal imaginable

(Place a mark on the scale above)

h. La satisfaction que vous obtenez de votre travail au regard des efforts fournis :

aucune satisfaction — satisfaction maximale imaginable

(Place a mark on the scale above)

i. D'investissement dans votre travail :

aucun investissement — investissement maximal imaginable

(Place a mark on the scale above)

j. Addiction au travail :

aucune addiction — addiction maximale imaginable

(Place a mark on the scale above)

k. D'anxiété :

aucune anxiété — anxiété maximale imaginable

(Place a mark on the scale above)

l. D'humeur :

humeur la pire imaginable — humeur la meilleur imaginable

(Place a mark on the scale above)

7) Nombre d’heures d’activité physique par semaine

____

8) Nombre d’heures assis(e) par jour :

____

9) Consommez-vous du tabac ?

○ Oui ○ Non

a. Si oui, précisez le nombre de cigarettes par jour:

____

10) Consommez-vous d'autres produits type vapotage etc :

○ Oui ○ Non

COMMENTAIRES

Ajouter des commentaires si vous le souhaitez: ____________________________________

Merci beaucoup pour votre participation.
